# Analysis of Fcγ receptor haplotypes in rheumatoid arthritis: *FCGR3A *remains a major susceptibility gene at this locus, with an additional contribution from *FCGR3B*

**DOI:** 10.1186/ar1847

**Published:** 2005-11-10

**Authors:** Ann W Morgan, Jennifer H Barrett, Bridget Griffiths, Deepak Subramanian, Jim I Robinson, Viki H Keyte, Manir Ali, Elizabeth A Jones, Robert W Old, Frederique Ponchel, Arthur W Boylston, R Deva Situnayake, Alexander F Markham, Paul Emery, John D Isaacs

**Affiliations:** 1 Institute of Molecular Medicine, Epidemiology and Cancer Research, University of Leeds, St James's University Hospital, Beckett Street, Leeds, LS9 7TF, UK; 2 Academic Unit of Musculoskeletal Disease, University of Leeds, Chapel Allerton Hospital, Chapeltown Road, Leeds, LS7 4SA, UK; 3 Department of Biological Sciences, University of Warwick, Coventry, CV4 7AL, UK; 4 City Hospital, Birmingham, Sandwell and West Birmingham Hospitals, NHS Trust, City Hospital Site, Dudley Road, Birmingham, B18 7QH, UK; 5 School of Clinical Medical Sciences (Musculoskeletal Research Group) University of Newcastle-Upon-Tyne, Framligton Place, Newcastle-Upon-Tyne, NE2 4HH, UK

## Abstract

The Fcγ receptors play important roles in the initiation and regulation of many immunological and inflammatory processes, and genetic variants (*FCGR*) have been associated with numerous autoimmune and infectious diseases. The data in rheumatoid arthritis (RA) are conflicting and we previously demonstrated an association between *FCGR3A *and RA. In view of the close molecular proximity with *FCGR2A, FCGR2B *and *FCGR3B*, additional polymorphisms within these genes and *FCGR *haplotypes were examined to refine the extent of association with RA. Biallelic polymorphisms in *FCGR2A*, *FCGR2B *and *FCGR3B *were examined for association with RA in two well characterized UK Caucasian and North Indian/Pakistani cohorts, in which *FCGR3A *genotyping had previously been undertaken. Haplotype frequencies and linkage disequilibrium were estimated across the *FCGR *locus and a model-free analysis was performed to determine association with RA. This was followed by regression analysis, allowing for phase uncertainty, to identify the particular haplotype(s) that influences disease risk. Our results reveal that *FCGR2A, FCGR2B *and *FCGR3B *were not associated with RA. The haplotype with the strongest association with RA susceptibility was the *FCGR3A–FCGR3B *158V-NA2 haplotype (odds ratio 3.18, 95% confidence interval 1.13–8.92 [*P *= 0.03] for homozygotes compared with all genotypes). The association was stronger in the presence of nodules (odds ratio 5.03, 95% confidence interval 1.44–17.56; *P *= 0.01). This haplotype was also more common in North Indian/Pakistani RA patients than in control individuals, but not significantly so. Logistic regression analyses suggested that *FCGR3A *remained the most significant gene at this locus. The increased association with an *FCGR3A–FCGR3B *haplotype suggests that other polymorphic variants within *FCGR3A *or *FCGR3B*, or in linkage disequilibrium with this haplotype, may additionally contribute to disease pathogenesis.

## Introduction

Rheumatoid arthritis (RA) is a heterogeneous disease characterised by a chronic, fluctuating, peripheral, symmetrical and erosive polyarthritis. It has been reported throughout the world, with a prevalence rate of approximately 1% in most populations [1]. Persistent synovial inflammation leads to progressive joint destruction, which in turn produces deformity and significant disability. In addition, RA is a systemic disease and some patients develop subcutaneous rheumatoid nodules, secondary Sjögren's syndrome, episcleritis and scleritis, interstitial lung disease, pericardial involvement, systemic vasculitis and Felty's syndrome. These extra-articular manifestations of RA appear to be rare in the absence of rheumatoid factor (RF), and IgG RF titres correlate with articular disease severity and with the extra-articular manifestations of RA [2]. In addition to RF, many other IgG autoantibodies are found in RA, most notably anti-cyclic citrullinated peptide [3] and anti-type II collagen antibodies [4].

The Fcγ receptors (FcγRs), which bind these IgG autoantibodies and IgG-containing immune complexes, have been shown to play important roles in the initiation and regulation of many immunological and inflammatory processes. In humans, there are three classes of FcγRs (I-III) encoded by eight genes, which produce at least 15 different membrane-bound and soluble isoforms that vary in their cellular distribution and affinity for different IgG isotypes. This molecular and expression diversity restricts specific biological properties to certain cell types. Activating (FcγRIIa, FcγRIIIa and FcγRIIIb) and inhibitory (FcγRIIb) FcγRs are frequently coexpressed on the same cell, thus providing a means for regulating signalling thresholds [5,6]. Furthermore, the absolute level of receptor expression is modulated by proinflammatory and anti-inflammatory cytokines [7]. Activating functions include uptake and clearance of immune complexes (complement dependent and independent mechanisms), activation of phagocytes (trigger the oxidative burst, cytotoxic granule and cytokine release), antigen presentation and antibody-dependent cellular cytotoxicity [6]. Conversely, FcγRIIb contains an inhibitory motif in the cytoplasmic tail and abrogates cellular activation. FcγRIIb may also play a role in maintaining peripheral B cell tolerance and prevention of autoimmunity [8]. Single and multiple FcγR knockout mouse models have demonstrated that the balance between activating and inhibitory FcγRs influences the development of both immune complex-mediated and collagen-induced arthritis [9,10].

Polymorphic variants that increase the expression or affinity of these IgG receptors or that enhance their ability to bind specific IgG isotypes may therefore play an important role in determining the severity and persistence of inflammation to IgG (auto)antibodies and immune complexes in RA. Our previous studies have supported an association between *FCGR3A *and RA. The higher affinity *FCGR3A*-158V allele was associated with an increased susceptibility to RA, with homozygotes demonstrating a 1.5-fold to twofold increased risk for RA and a twofold to fourfold increase in nodules [11,12]. In keeping with several other RA susceptibility genes, this association has not been replicated in all populations [13-18]. There was a trend toward an increased frequency of the *FCGR3A*-158V allele in Norwegian and Dutch RA patients [16,18], with a skewing toward the *FCGR3A*-158F allele in Spanish, Japanese and Indian populations [13-15,17]. Many reasons for the lack of reproducibility of association studies have been proposed and include differences in the design, power and accuracy of genotyping strategies in the various studies. The apparently conflicting results may also be a consequence of a genuine difference in the genetic and environmental susceptibility factors according to the precise ethnic group or disease phenotype under investigation. Nonreplication may thus indicate a real biological difference between populations. Alternatively, if *FCGR3A *is in linkage disequilibrium with the true RA-susceptibility locus then, because the extent of linkage disequilibrium varies between different populations, the association may only exist in certain populations and contribute to nonreplication of findings [12,19].

*FCGR3A *lies in a 200 kilobase *FCGR *gene cluster on 1q22-23 in close molecular proximity to *FCGR2A*, *FCGR3B *and *FCGR2B *[20]. Functional single nucleotide polymorphisms (SNPs) in the latter genes have been investigated as genetic susceptibility and severity factors in multiple infectious and autoimmune diseases [5,6]. We therefore undertook additional genotyping in our original RA cohorts and examined SNPs in *FCGR2A*, *FCGR2B *and *FCGR3B*; examined the extent of linkage disequilibrium at this locus; and analyzed *FCGR *haplotypes for association with disease in order to investigate the possibility that there are other RA susceptibility variants at this locus. We demonstrate an increased association with a *FCGR3A–FCGR3B *haplotype, which suggests that other polymorphic variants within *FCGR3A *or *FCGR3B*, or in linkage disequilibrium with this haplotype may additionally contribute to disease pathogenesis.

## Materials and methods

### Rheumatoid arthritis patients and control individuals

This was an allelic association study conducted to examine *FCGR2A*, *FCGR2B *and *FCGR3B*, and *FCGR *haplotypes in two well characterized RA cohorts in which an association between *FCGR3A *and RA was previously identified [11]. The recruitment and clinical characteristics of these two RA and control populations, resident in Birmingham, UK have previously been described [11,21]. They comprise 294 UK Caucasian individuals (150 RA patients and 144 healthy control individuals) and 256 North Indian/Pakistani individuals (126 RA patients and 130 healthy control individuals). Ethical approval was obtained from the respective local research ethics committees.

### Elucidation of the *FCGR *gene order

Computational assemblies of 1q23 at the National Center for BioInformatics [22], Ensembl [23] and Oak Ridge National Laboratory [24] websites resulted in several variations in *FCGR *gene order, thus necessitating the construction of an electronic contig. Genomic exon sequences of the class II (*FCGR2A*, *FCGR2B *and *FCGR2C*) and class III (*FCGR3A *and *FCGR3B*) genes were aligned to enable identification of homologous regions and specific nucleotides that distinguished the *FCGR *genes. All available sequence data containing the class II and III *FCGR *genes was identified by performing BLAST (basic local alignment search tool) sequence homology searches at the National Centre for BioInformatics [22] using five 30-base-pair homologous regions from each receptor class. The bacterial artificial chromosome (BAC) sequence fragments were primarily aligned by the identification of complete gene sequences and overlapping sequence data from neighbouring BACs, and the final assembly was facilitated by utilizing published restriction enzyme maps of this locus [20,25].

### *FCGR2B *sequence analysis

At the time when this study was performed, several putative polymorphic variants of *FCGR2B *had been identified in a cDNA library [26], but these had not been substantiated by sequencing genomic DNA. These included a 2-base-pair deletion at positions 208–210 at the start of exon 3, a G→A substitution at position 685 in exon 4, a functionally significant T→G substitution at position 855 in exon 6 [27] and a G→A substitution at position 1206 in the 3'-untranslated region (UTR). Two potential polymorphic sites (rs844 and rs1043) in STS accession G06355 (UniSTS:73835) were also identified from the reference SNP database, which corresponded to the 3'-UTR of *FCGR2B*.

The genomic sequence alignments of the class II *FCGR *genes (*FCGR2A, FCGR2C *and *FCGR2B*), generated as described above, were used to design *FCGR2B*-specific PCRs to facilitate direct sequencing of exons 3 and 6 and the 3'-UTR. The primer sequences and annealing temperatures of the different PCR reactions are shown in Table 1. Briefly, 20 μl PCRs were performed using 100 ng DNA, 200 nmol/l of each primer, 40 μmol/l each of 4 dNTPs, 1.5 mmol/l MgCl_2 _and 0.5 units of Taq DNA polymerase (Promega, Southampton, UK). The PCR reaction was performed in 30 individuals using a Techne Genius PCR machine (Techne [Cambridge] Ltd, Ducksford, Cambridge, UK) and the PCR conditions were 95°C for 5 minutes followed by 38 cycles of 95°C for 30 s, annealing temperature for 60 s and 72°C for 60 s, with a final extension step of 72°C for 10 minutes. Fluorescent automated cycle sequencing of the PCR products was performed using a dRhodamine terminator reaction kit (PE Biosystems, Warrington, UK). Electrophoresis was performed on polyacrylamide gels using the ABI PRISM^® ^377 DNA Sequencer (Applied Biosystems, Foster City, CA, USA) and the sequence analyzed utilizing ABI PRISM^® ^377 sequencing software.

### *FCGR *genotyping

#### *FCGR2A*

The *FCGR2A*-131H/R functional polymorphism has a G→A substitution at nucleotide 519, which results in a switch from arginine (R) to histidine (H) at amino acid position 131 in the immunoglobulin-binding domain [28]. Genotyping was performed using a nested amplification refractory mutation system (ARMS) PCR approach. A 322 bp PCR product was amplified (30 cycles) using a combination of previously published *FCGR2A*-specific primer sequences (PCR1 and 4INM) [28]. A 1:500 dilution served as a template for two separate nested ARMS PCRs that utilized 400 nmol/l of the published IIA-R and IIA-H primers [29]. The 322 bp product served as a positive control and a 246 bp product indicated the presence of either the *FCGR2A*-131H or R allele, according to the ARMS primer used. Specific amplification of *FCGR2A*, rather than the highly homologous *FCGR2B *and *FCGR2C*, and the +519 SNP, was confirmed in 40 individuals by direct sequencing.

#### *FCGR2B*

The *FCGR2B-*1206G/A polymorphism was genotyped using a nested RFLP assay. The 330 bp *FCGR2B*-specific sequencing PCR product (see above) was diluted 1:200 and served as a template for a second PCR that used IIB-UTRSF and a mutated reverse primer (IIB-UTRGR), which introduced an allele-specific *Hae*III restriction site. The resultant PCR product was incubated at 37°C for 1 hour with 6U *Hae*III (Promega) and the products visualized using a 3.5% agarose gel.

#### *FCGR3A*

Additional DNA samples that had not yielded reliable results on direct sequencing [11] were genotyped using our single-stranded conformational polymorphism assay [12] and were included in the haplotype analysis.

#### *FCGR3B*

The functional neutrophil antigen (NA)1 and NA2 alleles of *FCGR3B *were genotyped using minor modifications to a previously published ARMS PCR assay, which included both allele-specific primers and a distinct internal control [30]. The NA1 assay included the two ARMS primers IIIB-NA1F and IIIB-NA1R, and an internal control (fragment of *FCGR3A *and *FCGR3B) *was amplified by IIIB-NA1PF and IIIB-NA1PR. The NA2 assay similarly included the IIIB-NA2F and IIIB-NA2R ARMS primers and internal control primers IIIB-NA2PR and IIIB-NA2PR from the gene *MCSD1*.

### Statistical analyses

Statistical analyses were performed using the Stata statistical software (Stata Statistical Software, release 8.0; Stata Corporation, College Station, TX, USA) unless otherwise stated. Hardy–Weinberg equilibrium was investigated in each control population using a goodness-of-fit test to check whether the observed pattern of genotype frequencies was consistent with expectations. Allele and genotype frequencies were compared using 2 × 2 and 3 × 2 contingency tables, respectively. Nodules are only rarely present during the early stages of RA and their absence does not indicate that they may not develop in the future. The control population was therefore felt to be the most appropriate reference group for analysis of the subgroup with nodular RA [31].

Haplotype frequencies were estimated pair-wise across the *FCGR *locus using the Estimating Haplotypes PLUS (EHPLUS) program [32]. A pair-wise measure of linkage disequilibrium (*D*') was also calculated for each pair of *FCGR *genes. Association with disease was tested for by comparing the haplotype frequencies estimated from cases and controls separately with estimates based on the combined sample, using a likelihood ratio test. A permutation procedure implemented in the EHPLUS program was used to assess statistical significance based on 1,000 permutations [32].

If an individual is heterozygous at two loci, then the phase (for example, which variants are inherited from the same parent) is unknown. Association of *FCGR3A–FCGR3B *haplotypes with RA was investigated further using the haplotype trend regression (HTR) approach proposed by Zaykin and coworkers [33] for dealing with uncertain phase. In this method logistic regression can be used to predict disease status from an individual's haplotypes; where these are not known with certainty, all haplotypes consistent with the genotypes are included as predictors, weighted by their probabilities. This approach estimates the effect on risk for each haplotype, assuming that each of the individual's two haplotypes can have an independent effect. Stepwise regression analyses were also used to investigate the joint effect of the *FCGR *genes [34].

## Results

### Chromosomal order of the *FCGR *genes

The *FCGR *genes were located on three BAC clones: RP11-474I16 (EMBL: AL359541) contained *FCGR2B*; RP11-25I17 (EMBL: AC021370) contained *FCGR2B*, *FCGR2C*, *FCGR3A *and *FCGR3B*; and the final clone RP11-5K23 (EMBL: AC013307) contained *FCGR3A *and *FCGR2A*. The complete genomic sequence of each *FCGR *(with the exception of the 5' part of *FCGR2C*) was identified on these BAC clones. Alignment of the sequence fragments demonstrated the *FCGR *gene order from centromere to telomere at chromosome 1q23 as *FCGR2A*, *FCGR3A*, *FCGR2C*, *FCGR3B*, *FCGR2B*. Similar work is in agreement with this gene order [35].

### *FCGR2B *sequencing

The rs844 G→A substitution was confirmed and was identical to that described previously at position 1206 [26], and this SNP was therefore designated *FCGR2B*-1206G/A. No further polymorphisms were identified in exons 3 or 6 or the 3'-UTR of *FCGR2B *in 30 control individuals and RA patients.

### Association of *FCGR2A*, *FCGR3B *and *FCGR2B *with rheumatoid arthritis

Genotyping was complete on 274 Caucasian individuals (147 cases and 127 controls) and 249 North Indian/Pakistani individuals (122 cases and 127 controls).* FCGR2A*, *FCGR3A *and *FCGR2B *were in Hardy–Weinberg equilibrium in both control groups. *FCGR3B *was not in Hardy–Weinberg equilibrium (for the UK Caucasian group: *P *= 0.01; for the North Indian/Pakistani group: *P *= 0.002). We subsequently sequenced more than 200 individuals with 100% agreement with our genotyping assays to exclude genotyping error as an explanation.

No significant differences in the allele or genotype distributions were seen for *FCGR2A*, *FCGR3B*, or *FCGR2B *in either RA group compared with controls (Table 2). The results for *FCGR3A *in our expanded UK Caucasian cohort were consistent with our previous findings [11]. Homozygosity for the *FCGR3A*-158V allele demonstrated a trend toward and association with RA (odds ratio [OR] 2.1, 95% confidence interval [CI] 1.0–4.7; *P *= 0.06) and significant association with nodular RA (OR 4.3, 95% CI 1.5–12.3; *P *= 0.005).

### Linkage disequilibrium at the *FCGR *genetic locus

There was evidence of weak linkage disequilibrium (*D' *= 0.30, *P *= 0.01) between *FCGR2A *and *FCGR3A *in the UK Caucasian but not the North Indian/Pakistani control populations. Highly significant linkage disequilibrium was seen between *FCGR3B *and *FCGR2B *in both populations (UK Caucasian: *D' *= -0.68, *P *= 0.0001; North Indian/Pakistani:*D' *= -0.52, *P *= 0.0001). The negative *D' *values indicate linkage disequilibrium between the common allele of one gene and the rare allele of the second gene. No significant linkage disequilibrium was detected between *FCGR3A *and *FCGR3B *in either ethnic group, although *D' *= 0.40 in the UK Caucasian group (Table 3).

### Association of *FCGR *haplotypes with rheumatoid arthritis

The distributions of two locus *FCGR *haplotypes were compared between the RA cohorts and their control populations, with a difference approaching statistical significance for *FCGR3A–FCGR3B *(Table 4). Compared with the control frequency of 24%, the *FCGR3A–FCGR3B *158V-NA2 haplotype was found at increased frequency in UK Caucasian RA patients (31%) and even higher frequency in those with nodular RA (37%).

From the HTR analysis of *FCGR3A*–*FCGR3B *haplotypes, the 158V-NA2 haplotype was found to have a significant effect on the risk for RA in UK Caucasians (OR 1.77, 95% CI 1.09–2.87; *P *= 0.02), taking the most common haplotype (158F-NA2) as baseline (Table 5). The effect was stronger in the small subgroup of UK Caucasian individuals with nodules (OR 2.51, 95% CI 1.15–5.49; *P *= 0.02).

These are estimates of the effect of each haplotype, assuming that each of the individual's two haplotypes has an independent effect on risk with a combined multiplicative effect. The effect of the 158V-NA2 haplotype was found to be largely confined to those with two copies of this haplotype (data not shown). To estimate the effect under a recessive model, homozygosity for this haplotype was compared in the control population (frequency 4%) with the total RA population (11%), giving an OR of 3.18 (95% CI 1.13–8.92; *P *= 0.03) when comparing homozygotes with all others. Again, the effect of homozygosity was stronger in those with nodular RA (frequency 16%; OR 5.03, 95% CI 1.44–17.56; *P *= 0.01).

For the North Indian/Pakistani cohort the same haplotype was found to be at increased frequency in RA patients compared with controls (OR 1.52, 95% CI 0.82–2.80, from the HTR analysis) but the difference was not statistically significant (*P *= 0.19; Table 5). Homozygosity for this haplotype was seen in approximately 4% of the RA and 1.5% of the control population.

### Stepwise logistic regression analyses of *FCGR3A *and *FCGR3B *in the Caucasian group

Considering each locus separately, *FCGR3A *is associated with RA in Caucasians [11] but *FCGR3B *is not (Table 2). However comparing the model containing both genotypes with the model containing *FCGR3A *only, there is an improved fit with inclusion of *FCGR3B *(χ^2 ^= 6.27, 2 degrees of freedom, from likelihood ratio test; *P *= 0.04). When the cohort with nodules was examined, the inclusion of *FCGR3B *did not significantly improve the model (*P *= 0.22).

### Contribution of *FCGR *haplotypes and shared epitope alleles in rheumatoid arthritis susceptibility

One advantage of the HTR framework for analysis of haplotypes is that other factors can be included in the model. The analysis was repeated including the RA-associated 'shared epitope' (SE) alleles (positive or negative) in the models. As expected, the SE itself was still highly predictive of RA in these models (OR 3.16, 95% CI 1.75–5.71 in UK Caucasians; OR 3.94, 95% CI 2.17–7.18 in the North Indian/Pakistani group). There was evidence of a multiplicative joint effect between SE and the *FCGR *haplotypes, consistent with both of these two genetic factors contributing to the risk for disease. Thus, the risk for RA in SE-positive UK Caucasian individuals homozygous for the *FCGR3A*–*FCGR3B *158V-NA2 haplotype is increased tenfold compared with those with other *FCGR3 *genotypes who are SE negative.

## Discussion

Haplotype analyses have started to assume an increased importance in genetic studies of human disease because they can be more informative in their ability to identify unique chromosomal segments that are likely to harbour disease predisposing genes. They may also provide additional evidence for the presence of further unidentified polymorphic variants that are the true disease-susceptibility variants [36]. We have demonstrated an increased level of association between *FCGR3A–FCGR3B *haplotypes and RA compared with *FCGR3A *alone. The effects were stronger in the subset of RA patients with nodules. The two-locus haplotype showing the strongest association with RA susceptibility in each group was the *FCGR3A–FCGR3B *158V-NA2 haplotype. UK Caucasian individuals who were homozygous for this haplotype were estimated to be at threefold risk for disease compared with all others (OR 3.18, 95% CI 1.13–8.92; *P *= 0.03), and this analysis does not depend on inference of uncertain phase because individuals that are homozygous for a haplotype are unambiguously identified by their genotype. The relative importance of the *FCGR3A*-158V and *FCGR3B*-NA2 polymorphic variants were assessed further using stepwise regression analyses [34]. These analyses showed that, although only *FCGR3A *has been shown to be associated with RA [11], the model including both loci provided an improved fit for RA susceptibility, but not necessarily so for the development of nodules. In the North Indian/Pakistani population there was a 6% increase in the *FCGR3A*–*FCGR3B *158V-NA2 haplotype, but this failed to reach statistical significance.

Several methods have been proposed for the analysis of such data in the absence of family data and the consequent presence of phase uncertainty. The HTR method we have chosen to use has various advantages. The method uses all the data, including that from individuals with uncertain phase. Because the data are analyzed in a regression framework, the usual regression diagnostics are available, other factors can be included in the model and tests for interaction can be performed. A potential disadvantage of the method is that weights used in the regression analysis are based on estimated haplotype frequencies, and the uncertainty inherent in the estimates is ignored in the model. This leads to an anti-conservative test and could lead to false-positive results. However, Stram and coworkers [37] evaluated the method in comparison with other more sophisticated approaches and found it to perform well in most situations.

Haplotype frequencies were estimated using the expectation-maximization algorithm, which has been shown to perform well, even in the presence of some departure from Hardy–Weinberg equilibrium [38]. Errors due to sampling are generally of much greater concern than inaccuracies due to the estimation process.

We acknowledge that none of our results are highly statistically significant when a consideration is made for multiple tests. However, the consistent pattern of results, taken in conjunction with prior findings in relation to these genes and their known biological functions in humans and mice, gives additional credence to them. Replication of these findings in other populations will ultimately be required.

The FcγRs play important roles in the initiation and propagation of many different immunological and inflammatory processes. Consequently, they may act as susceptibility factors for RA through a variety of mechanisms. *FCGR3A *was the most significant gene in this study, and we have previously discussed the role that this receptor may play in RA pathogenesis [11,12]. In humans, FcγRIIIa is expressed on natural killer cells, macrophages, γδ T cells, a subset of monocytes and cultured mast cells [5,6]. Higher levels of FcγRII and FcγRIII expression have been demonstrated in synovial biopsy specimens from RA patients compared with control individuals [39]. Similarly, an increase in the expression level and proportion of circulating FcγRIIIa-positive monocytes has been observed in RA and may correlate with disease activity [40,41]. In addition, *in vitro *derived macrophages from RA patients expressed more FcγRII and FcγRIIIa, and released higher levels of tumour necrosis factor-α and matrix degrading enzymes in response to heat-aggregated IgG [39] compared with controls. These findings are supportive of our own work whereby the higher affinity genetic variant of *FCGR3A *may sensitize FcγR-bearing cells to IgG-containing immune complexes. FcγRIIIa may also play an important role delivering (auto)antigens, and activation and maturation signals to dendritic cells [42]. This may provide an explanation for the over tenfold increased risk for RA in SE-positive Caucasian individuals homozygous for the *FCGR3A*–*FCGR3B *158V-NA2 haplotype, which has been a consistent finding by a number of groups [11-13,15,18].

FcγRIIIb is selectively expressed on neutrophils and eosinophils, and has a low affinity for IgG. It is linked to the membrane by a glycosylphosphatidylinositol anchor and does not appear to associate with the known transmembrane adapter molecules [5,6]. However, FcγRIIIb appears to interact with FcγRIIa in the phagocytosis of immune complexes and subsequent cellular activation, with signalling being mediated through the ITAM (immunoreceptor tyrosine-based activation motif) of FcγRIIa [43,44].* FCGR3B *has two common polymorphic forms, namely NA1 and NA2, which differ in five nucleotides that produce four amino acid differences. This alters the number of glycosylation sites, and neutrophils from individuals homozygous for the *FCGR3B*-NA2 allele have been found consistently to exhibit lower levels of phagocytosis than *FCGR3B*-NA1 homozygotes [45]. This polymorphism has important biological consequences, especially in the development of blood transfusion reactions, autoimmune neutropenias and the severity of renal disease in systemic vasculitis [6,46]. Individuals with duplications and deletions of *FCGR3B *have been reported [30,47], with the estimated frequency of the *FCGR3B *deletion being 0.001–0.08 in various Caucasian populations [48]. Standard genotyping assays, as performed in the present study, do not allow a calculation of the gene copy number. This may provide an explanation for a failure of our control populations to conform to Hardy–Weinberg equilibrium and the previously reported non-Mendelian segregation in some Caucasian families [49].

FcγRIIb plays a crucial role in the regulation of antibody production and susceptibility to several spontaneous and induced murine autoimmune diseases [50-52]. We found no evidence of an association between *FCGR2B*- or *FCGR2B*-containing haplotypes and RA in our cohorts, unlike previous observations in a Japanese cohort in which an alternative SNP in *FCGR2B *was investigated [15].

## Conclusion

There is good data that FcγRs may be critical modulators of inflammation within the synovium and that subtle changes in either expression or structure of these receptors may influence both the susceptibility to RA and the development of nodules. The analyses performed in this study have strengthened our original observation that the *FCGR *genetic locus is associated with RA, particularly in a UK Caucasian population with nodular disease. Our haplotype data, together with the stepwise regression analysis, suggest that additional polymorphic variants within *FCGR3A *or in linkage disequilibrium with the *FCGR3A–FCGR3B *158V-NA2 haplotype may contribute to RA pathogenesis.

## Abbreviations

ARMS = amplification refractory mutation system; BAC = bacterial artificial chromosome; bp = base pairs; BLAST = basic local alignment search tool; CI = confidence interval; FcγR = Fcγ receptor; HTR = haplotype trend regression; NA = neutrophil antigen; OR = odds ratio; PCR = polymerase chain reaction; RA = rheumatoid arthritis; RF = rheumatoid factor; SE = shared epitope; SNP = single nucleotide polymorphism; UTR = untranslated region.

## Competing interests

The authors declare that they have no competing interests.

## Authors' contributions

AWM participated in the design of the study, undertook all database searches, oversaw all aspects of the laboratory work, analyzed the data and prepared the manuscript. JHB gave additional statistical support and performed the haplotype analysis. BG, RDS and PE participated in the collection of clinical data and the recruitment of patients into the study. DS, JR and VK undertook some of the genotyping assays on DNA prepared in the laboratory of EAJ and RWO, who participated in the original design of the study. FP and MA gave invaluable advice during the retrieval of sequence data from the public databases and during the optimization of some genotyping assays. AWB, AFM, PE and JDI participated in the design of the study, interpretation of the results and writing of the final manuscript.

## Figures and Tables

**Table 1:** Primer pairs used to amplify specific *FCGR *sequencing templates

Gene	Forward primer	Sequence	Reverse primer	Sequence	Annealing temperature (°C)
*Sequencing primers*					
*FCGR2A*	IIA-SF	dGGAGAAACCATCATGCTGAG	IIA-SR	dCAATTTTGCTGCTATGGGC	52
*FCGR2B *(E3)	IIB-E3SF	dGGCATCTCAAGCTCC	IIB-E3SR	dAGAGTCACAGAGTCCTCC	58
*FCGR2B *(E6)	IIB-E6SF	dCCCATCCAACCCTGG	IIB-E6SR	dGGCAGATTCCTCAGCAAATCA	56
*FCGR2B *(3'UTR)	IIB-UTRSF	dTGGGGAGGACAGGGAGAT	IIB-UTRSR	dATCACTTTTAATGTGCTGGTAGAGG	63
Genotyping primers					
*FCGR2A*	PCR1	dGGAGAAACCATCATGCTGAG	4INM	dCAATTTTGCTGCTATGGGC	52
	IIA-R	dAATCCCAGAAATTCTCCCG	IIA-H	dAATCCCAGAAATTCTCCCA	
*FCGR3B*-NA1	IIIB-NA1F	dCAGTGGTTTCACAATGTGAA	IIIB-NA1R	dATGGACTTCTAGCTGCACCG	54
	IIIB-NA1PR	dGTCTCTTTCTGCTTGGTGATGG	IIIB-NA1PR	dTTTTCCCCTCTAAACTGGG	
*FCGR3B*-NA2	IIIB-NA2F	dCTCAATGGTACAGCGTGCTT	IIIB-NA2R	dCTGTACTCTCCACTGTCGTT	62
	IIIB-NA2PR	dCTGGCTTGCTGATGAAGATAC	IIIB-NA2PR	dGTAACGCTTNGGCACCACC	
*FCGR2B*	IIB-UTRSF	dTGGGGAGGACAGGGAGAT	IIB-UTRGR	dCAGAAGGTGCAGTCGG*C*	50

The mutation inserted into the *FCGR2B *reverse nested primer (IIB-UTRGR) is shown in italics. This introduced an allele-specific *Hae*III restriction site (GGCC) in the presence of the G but not the A allele.

**Table 2:** Genotype frequencies in UK Caucasian and North Indian/Pakistani rheumatoid arthritis patients and healthy controls

Gene	Genotype	UK Caucasian					North Indian/Pakistani		
		
		Control (*n *= 129)	RA (*n *= 147)	*P*	Nodular RA (*n *= 37)	*P*	Control (*n *= 128)	RA (*n *= 123)	*P*
*FCGR2A*-131	RR	39 (0.31)	40 (0.28)	0.81	7 (0.19)	0.36	25 (0.19)	30 (0.25)	0.15
	RH	59 (0.47)	72 (0.49)		21 (0.58)		66 (0.52)	48 (0.39)	
	HH	28 (0.22)	34 (0.23)		8 (0.22)		37 (0.29)	44 (0.36)	
*FCGR3A*-158	FF	68 (0.48)	59 (0.39)	0.15	12 (0.31)	0.02	63 (0.49)	48 (0.38)	0.21
	FV	61 (0.43)	69 (0.46)		17 (0.45)		57 (0.44)	66 (0.52)	
	VV	12 (0.09)	22 (0.15)		9 (0.24)		9 (0.07)	12 (0.10)	
*FCGR3B*-NA	22	48 (0.37)	55 (0.38)	0.13	16 (0.43)	0.28	49 (0.39)	47 (0.39)	0.25
	21	72 (0.56)	71 (0.48)		16 (0.43)		45 (0.35)	53 (0.43)	
	11	9 (0.07)	21 (0.14)		5 (0.14)		33 (0.26)	22 (0.18)	
*FCGR2B*-1206	GG	56 (0.48)	67 (0.46)	0.96	16 (0.43)	0.19	61 (0.48)	58 (0.47)	0.98
	GA	50 (0.42)	63 (0.43)		13 (0.35)		54 (0.43)	54 (0.44)	
	AA	12 (0.10)	16 (0.11)		8 (0.22)		11 (0.09)	11 (0.09)	

Values are expressed as number (%). RA, rheumatoid arthritis.

**Table 3:** Pair-wise linkage disequilibrium measures (*D'*) calculated from the control groups in the two populations

	*FCGR2A*	*FCGR3A*	*FCGR3B*
*FCGR3A*	**0.30 **(0.18)		
*FCGR3B*	0.05 (0.23)	**-0.40 **(-0.21)	
*FCGR2B*	-0.10 (0.05)	0.00 (**-0.34**)	**-0.68 **(**-0.52**)

Shown are *D' *measures for the UK Caucasian (and North Indian/Pakistani) populations. Values with a magnitude of 0.3 and higher highlighted in bold.

**Table 4:** Estimated pairwise haplotype frequencies in rheumatoid arthritis patients and healthy controls

Genes	Haplotype	UK Caucasian					North Indian/Pakistani		
		
		Control	RA	*P*^a^	Nodular RA	*P*	Control	RA	*P*
*FCGR2A-FCGR3A*	131R-158F	0.43	0.41	0.25	0.32	0.13	0.34	0.30	0.46
	131R-158V	0.11	0.11		0.16		0.11	0.15	
	131H-158F	0.27	0.22		0.22		0.36	0.34	
	131H-158V	0.19	0.26		0.30		0.19	0.21	
*FCGR3A-FCGR3B*	158F-NA2	0.42	0.31	0.08	0.27	0.07	0.38	0.36	0.33
	158F-NA1	0.28	0.32		0.27		0.34	0.29	
	158V-NA2	0.24	0.31		0.37		0.19	0.25	
	158V-NA1	0.06	0.06		0.09		0.10	0.11	
*FCGR3B-FCGR2B*	NA2-1206G	0.36	0.34	0.77	0.35	0.41	0.33	0.33	0.45
	NA2-1206A	0.29	0.27		0.30		0.24	0.28	
	NA1-1206G	0.31	0.33		0.26		0.37	0.37	
	NA1-1206A	0.04	0.06		0.09		0.06	0.03	

Pairwise haplotypes produced from four biallelic markers (*FCGR2A*-131H/R, *FCGR3A*-158F/V, *FCGR3B*-NA2/1 and *FCGR2B*-1206G/A) denoted in the order they occur at the *FCGR *locus. Thus, 131R-158F indicates a haplotype containing *FCGR2A*-131R and *FCGR3A*-158F alleles. ^a^Empirical *P *values obtained from a heterogeneity test statistic incorporated in the PM program after 1,000 permutations. RA, rheumatoid arthritis.

**Table 5:** Haplotype trend regression in rheumatoid arthritis patients and healthy controls for *FCGR3A-FCGR3B *haplotypes

Haplotype	UK Caucasian				North Indian/Pakistani	
	
	RA	*P*	Nodular RA	*P*	RA	*P*
158F-NA2	1 (baseline)	-	1 (baseline)	-	1 (baseline)	-
158F-NA1	1.62 (0.99–2.66)	0.05	1.58 (0.67–3.70)	0.29	0.96 (0.60–1.54)	0.85
158V-NA2	1.77 (1.09–2.87)	0.02	2.51 (1.15–5.49)	0.02	1.52 (0.82–2.80)	0.19
158V-NA1	1.44 (0.61–3.40)	0.41	2.00 (0.63–6.39)	0.24	1.22 (0.63–2.37)	0.55

Values are expressed as odds ratio (95% confidence interval). RA, rheumatoid arthritis.
